# Functional Conservation of the Small GTPase Rho5/Rac1—A Tale of Yeast and Men

**DOI:** 10.3390/cells13060472

**Published:** 2024-03-07

**Authors:** Linnet Bischof, Franziska Schweitzer, Jürgen J. Heinisch

**Affiliations:** AG Genetik, Fachbereich Biologie/Chemie, University of Osnabrück, Barbarastrasse 11, D-49076 Osnabrück, Germany; linnet.bischof@uni-osnabrueck.de (L.B.); fschweitzer@uni-osnabrueck.de (F.S.)

**Keywords:** Rac1, Rho5, molecular switch, oxidative stress, glucose signaling, mitochondria, apoptosis, NADPH oxidase

## Abstract

Small GTPases are molecular switches that participate in many essential cellular processes. Amongst them, human Rac1 was first described for its role in regulating actin cytoskeleton dynamics and cell migration, with a close relation to carcinogenesis. More recently, the role of Rac1 in regulating the production of reactive oxygen species (ROS), both as a subunit of NADPH oxidase complexes and through its association with mitochondrial functions, has drawn attention. Malfunctions in this context affect cellular plasticity and apoptosis, related to neurodegenerative diseases and diabetes. Some of these features of Rac1 are conserved in its yeast homologue Rho5. Here, we review the structural and functional similarities and differences between these two evolutionary distant proteins and propose yeast as a useful model and a device for high-throughput screens for specific drugs.

## 1. Introduction

All cells need to adjust properly to changing environmental conditions, such as the presence or absence of nutrients or hormones and temperature or pH shifts. Signaling cascades ensuring these proper responses in eukaryotic cells frequently employ small GTPases as molecular switches, which can alternate between an inactive, GDP-bound, and an active, GTP-bound, state. The latter enables their interaction with a broad range of downstream effector proteins. They thus regulate a plethora of cellular functions such as gene expression, cell cycle progression, vesicle transport, energy metabolism, and motility, with severe consequences of malfunctions for human health (see [1,2,3] and references therein). This is also true for the subfamily of Rho-type (for Ras homologue) GTPases, which comprises approximately 20 members in mammals [4] but only 6 in the model yeast *Saccharomyces cerevisiae* (i.e., Rho1 to Rho5 and Cdc42 [5]).

This review will focus on one family member, mammalian Rac1 (Ras-related C3 botulinum toxin substrate 1), and its yeast homologue Rho5 [6,7]. Moreover, as there were more than 8000 hits in PubMed (https://pubmed.ncbi.nlm.nih.gov/) on “Rac1 GTPase” as of 30 January 2024, we will further concentrate on the shared functions of the two GTPases in these evolutionary distant organisms when associating with and travelling to mitochondria. For a broader overview on other important roles of Rac1 and its fellow Rho-type GTPases in human health and disease, the reader is referred to several excellent reviews [1,2,3,8,9,10,11,12,13,14,15,16,17,18,19,20,21,22,23], the indicated selection being by no means exhaustive, with apologies to all authors inevitably left unacknowledged. The apology also extends to citations on details of Rac1 regulation, which only refer to a few recent original works that cover previous literature.

## 2. Comparison of Yeast Rho5 and Mammalian Rac1: Sequence, Structure, Regulation

**Figure 1:**
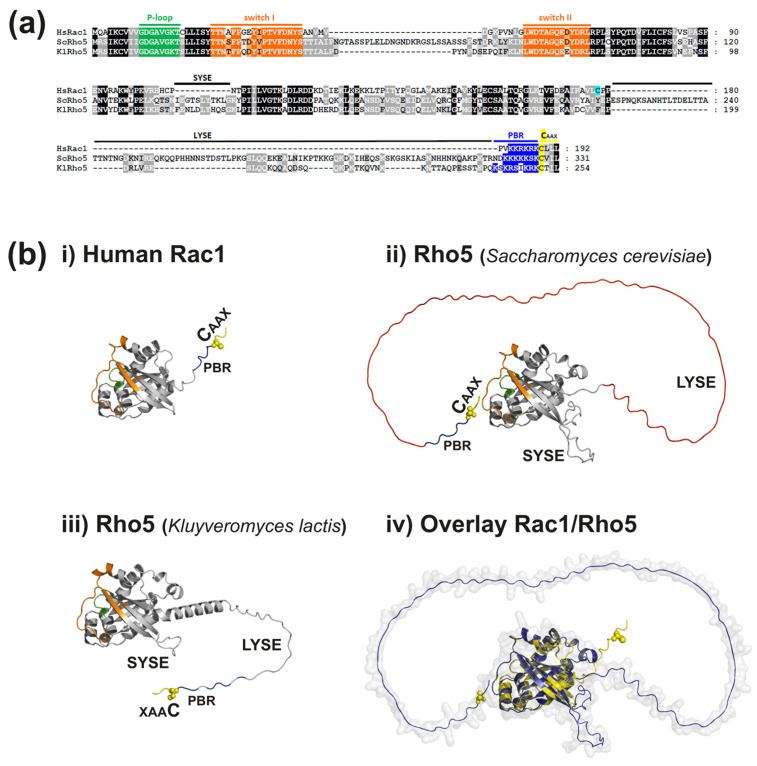
Structures of two yeast Rho5 proteins and human Rac1. (**a**) Alignments of primary sequences revealing conserved structural motives. Multiple sequence alignments are based on ClustalW, using the MUSCLE tool ([31]), and were graphically curated with GeneDoc version 2.5.000 (National Resource for Biomedical Supercomputing, Pittsburgh, PA, USA). Functional cysteine residues mentioned in the text are highlighted in yellow or light blue. (**b**) Structures of the human and yeast GTPases predicted by the AlphaFold program [32,33] and comparison of human Rac1 with Rho5 from *Saccharomyces cerevisiae*. Structures were obtained from the AlphaFold Protein Structure Database ([34], with accession numbers A0A816AYR0 for ScRho5, A0A5P2U790 for KlRho5, and P63000 for Rac1) and visualized using PyMOL (Schrödinger and DeLano, PyMOL, 2020; accessed on 20 December 2024 at http://www.pymol.org/pymol). In images (**i**–**iii**), the conserved switch I, switch II (both in orange), and P-loop sequences (green) are highlighted within the globular catalytic core. The C-terminal cysteine residues (yellow spheres) are modified by geranylgeranylation after removal of the AAX sequence (two aliphatic and a third, variable amino acid residue) during posttranslational modification. The preceding polybasic region (PBR) is shown in blue. The long and short yeast-specific extensions (LYSE and SYSE, respectively) are indicated for the two yeast homologues as basically unstructured strings, starting with a helix in *Kluyveromyces lactis*. For the overlay of human Rac1 with Rho5 from *S. cerevisiae* (structures **i** + **ii** presented in **iv**), the entire structures are depicted in yellow and blue, respectively.

Rho5 and Rac1 are small monomeric GTPases with a conserved domain structure. The domains comprise the switch I and switch II regions and a P-loop in the N-terminal part and the polybasic region (PBR) followed by a CAAX box (a cysteine followed by two aliphatic amino acid residues and a final variable residue) at the C-terminal end (Figure 1). While the switch regions modulate nucleotide binding within the P-loop, and thereby activation and interaction with effectors [24,25], the PBR and CAAX-box domains, after lipid modification of the latter, mediate the interactions with specific endomembranes, i.e., the intracellular localization and activation of the GTPases [26]. The PBR is preceded by a hypervariable region (HVR), which, as suggested by its name, differs considerably in its primary sequence between the various Rho-type GTPases. It is believed to mediate their association with and regulation of downstream effector proteins and thereby the specificity of the physiological response [27,28]. With regard to Rho5, this region is especially interesting, as it comprises a long yeast-specific extension (LYSE) of 98 amino acid residues (in the baker’s yeast *S. cerevisiae*) or 43 residues (in the milk yeast *Kluyveromyces lactis*) not present in mammalian Rac1 (Figure 1b; [29,30]). The extension is essential for proper physiological function, as neither its internal deletion from Rho5, nor the human Rac1 homologue rescues the mutant phenotypes in *S. cerevisiae*. Yet, a chimeric GTPase consisting of Rac1 fused to the extended C-terminus of Rho5 at least partially restores function in yeast, indicating that the short yeast-specific extension (SYSE) located between the two switch regions is of less functional importance (Figure 1b; [30]).

It should be noted that the LYSE region of Rho5 clearly distinguishes this protein from the other five Rho-type GTPases of *S. cerevisiae*, as this extension is found neither in Cdc42, nor in Rho1–Rho4 GTPases. With respect to Rac1/Rho5 homologues from other yeasts and related fungi, LYSE regions with completely different primary sequences are present in *Candida albicans* and *Ashbya gossypii*, but not in the homologues of *Neurospora crassa*, *Aspergillus nidulans*, *Yarrowia lipolytica*, or *Cryptococcus neoformans* (Figure S1). This offers the opportunity for species-specific regulation. As evident from the predicted structures shown in Figure 1b, the LYSE region constitutes an intrinsically disordered protein domain. Such sequences are typical for proteins involved in cellular signaling processes, as they can adapt to and interact with many proteins [35]. In fact, Rho5 was found to participate in various stress responses mediated by different signaling cascades, as discussed below in the section on its physiological functions. The intrinsically disordered domain could thus function as a hub for the detection of different environmental cues. Vice versa, assuming 0.36 nm for the length of a peptide bond, it would allow the globular part with the switch domains to extend approximately 35 nm into the cytosol from any endomembrane the GTPase is associated with by its lipid anchor, facilitating its access to soluble effector proteins.

**Figure 2:**
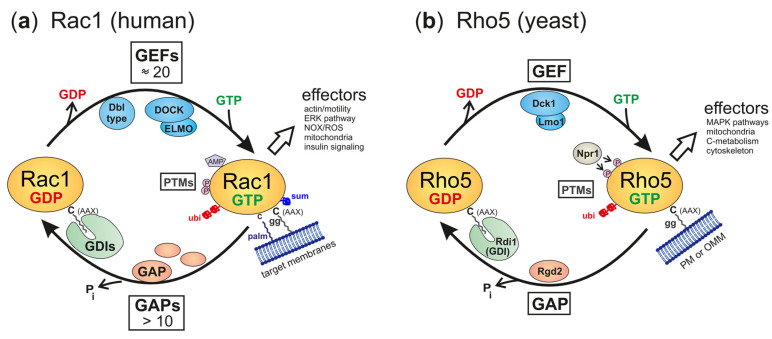
Regulatory circuits and ancillary proteins affecting the activation state of Rac1 (**a**) and Rho5 (**b**). The GTPases are shown in their inactive, GDP-bound state, and their active, GTP-bound, state. Ancillary proteins for the interconversion are GEFs (GDP/GTP exchange factors), GAPs (GTPase-activating proteins), and GDIs (GDP dissociation inhibitors), with the latter believed to shield the lipid modification to facilitate traveling through the hydrophilic cytosol. For human Rac1, a variety of GEFs and GAPs have been identified, with GEFs belonging to either the DOCK or the Dbl type (note that the DOCK/ELMO complex is depicted as an example of the former, as it coined the names of the yeast GEF subunits). PM = plasma membrane, OMM = outer mitochondrial membrane. Abbreviations for posttranslational modifications (PTMs) are P = phosphorylation, ubi = ubiquitinylation, sum = sumoylation, AMP = adenylation, palm = palmitoylation, gg = geranylgeranylation. See text for further details on the effectors listed.

One reason why the LYSE region may have conferred an evolutionary advantage to yeast Rho5 and is not required in its human homologue may lie in the diversity of their direct regulators (Figure 2).

Small GTPases from yeasts to humans are usually active in the GTP-bound state and assume an inactive conformation when it is hydrolyzed to the GDP-bound state. The interconversion between these states is facilitated by the help of ancillary proteins, namely, guanine nucleotide exchange factors (GEFs), which trigger the release of GDP to be substituted by GTP present in higher intracellular concentrations, and GTPase-activating proteins (GAPs), which promote the intrinsic hydrolytic activity towards bound GTP. In addition, GTPases may travel through the cytosol in association with GDP dissociation inhibitors (GDIs), which mask the lipid residue attached to the C-terminal cysteine (Figure 2a). In contrast to the original view that GDIs mediate the cytosolic transport of inactive GTPases, evidence from mammalian Rac1 indicates that GTPases can be stabilized in an active conformation by the interaction [36].

This general cycle of activation and inactivation applies to both Rho5 in yeast and Rac1 in humans. However, in yeast, only one dimeric GEF composed of Dck1 and Lmo1 is believed to activate Rho5, and Rgd2 has been proposed to act as its GAP (Figure 2b; [37,38]). By contrast, mammalian Rho-type GTPases can be activated by 80 different GEFs, more than 20 of which can act on Rac1 in different cell types and physiological conditions [10,39]. The Rho GEFs comprise two sub-families, with 69 monomeric Dbl-type and 11 dimeric DOCK-type members. They are matched by several inactivating GAPs (Figure 2a; reviewed in [8,40]). Clearly, this vast number of regulators provides a possibility for fine-tuning Rac1 activity that can hardly be matched by the interaction of the intrinsically disordered LYSE domain of Rho5 with effector proteins in the yeast environment.

Further regulation of GTPase activity, both for Rho5 and for Rac1, may be conferred by post-translational modifications, first of all by geranylgeranylation at the CAAX-box cysteine residue, but also by phosphorylation and ubiquitinylation (Figure 2; reviewed in [41] for fungi and in [16] for mammals). Lipidation at the carboxyterminal end enables Rho5 and Rac1 to associate with membranes, which is further enhanced by the electrostatic affinity between phospholipids and the PBR regions of the GTPases [26]. Rac1 can also be adenylated at the conserved tyrosine 32 residue [42], reversibly palmitoylated at cysteine 178 [43], or sumoylated at lysine residues within the PBR [44,45], all of which modulate its activity, stability, and/or subcellular localization, with no indications that these modifications occur in yeast Rho5, so far.

Originally, the plasma membrane was identified as the major compartment where the GTPases in fungi and mammalian cells localized and exerted their functions [46,47]. However, important physiological roles for Rac1 have subsequently been discovered in relation to its association with different endomembranes (reviewed in [48]) and will be briefly discussed in the following sections.

## 3. Intracellular Dynamics and Physiological Functions in Yeasts and Humans

**Figure 3:**
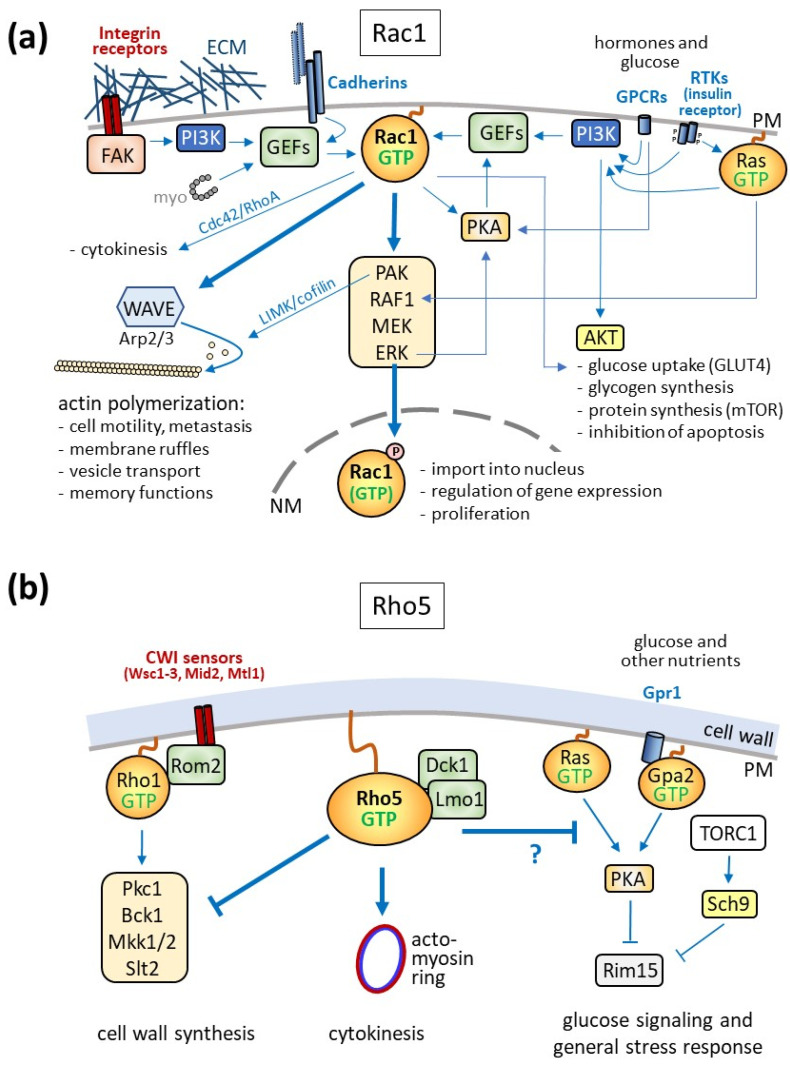
Physiological roles and signaling pathways controlled by Rac1 (**a**) and Rho5 (**b**). (**a**) General overview of pathways activating Rac1 and its downstream targets. Note that these pathways may be engaged differentially only in certain tissues and physiological conditions. Dbl-type GEFs can activate Rac1 upon interaction with non-muscle myosin (myo), which promotes cytokinesis in collaboration with the small GTPases Cdc42 and RhoA. Extracellular signals such as perturbations in the extracellular matrix (ECM, sensed by integrins and mediated by focal adhesion kinase, FAK), neighboring cells (sensed by cadherins), growth factors, and hormones (sensed by receptor tyrosine kinases, RTKs, or G-protein-coupled receptors, GPCRs) are perceived by receptors within the plasma membrane (PM) and lead to the activation of phosphoinositide-3-kinase (PI3K). The resulting phospholipids recruit GDP/GTP exchange factors (GEFs) for Rac1, which trigger the formation of its active GTP-bound state. One target of Rac1-GTP is the WAVE (WASP family verprolin-homologous) complex, promoting actin polymerization with the physiological consequences indicated. Another target of Rac1-GTP is a MAPK (mitogen activated protein kinase) cascade (box with PAK = p21-activated kinase, RAF1 = rat fibrosarcoma, MEK = MAP or ERK kinase, ERK = extracellular signal receptor kinase). PAK also regulates actin polymerization through the activation of LIM kinase (LIMK) and cofilin. ERK, at the lower end of the MAPK cascade, phosphorylates Rac1 and promotes its nuclear import and interaction with transcription factors. RTKs trigger the activation of Rac1 either through PI3K or, more indirectly, through the small GTPase Ras. Ras-GTP also enhances Rac1 signaling through the ERK cascade at the level of the MAPKKK RAF1. In addition to activating Rac1 GEFs, PI3K also leads to the activation of protein kinase B (AKT), which mediates glucose signaling with the physiological consequences indicated. GPCRs activate IP3K and thereby Rac1, which in turn participates in the regulation of the delivery of the glucose transporter GLUT4 to the plasma membrane. In addition, GPCRs trigger the production of cAMP by adenylate cyclase and thus activate PKA, which can activate specific Rac1 GEFs. (**b**) In yeast, Rho5 is attached to the plasma membrane under standard growth conditions. It is activated by the dimeric GEF Dck1/Lmo1 and works as a negative regulator of the MAPK cascade, which is triggered by the small GTPase Rho1 (activated by its monomeric GEF Rom2), leading to the activation of protein kinase C (Pkc1). This then activates the MAPK cascade consisting of the MAPKKK Bck1, a redundant pair of MAPKKs (Mkk1 and Mkk2), and the MAPK Slt2, which ultimately promotes cell wall remodeling. Rho5-GTP in the milk yeast *Kluyveromyces lactis* also promotes cytokinesis by interaction with the contractile actomyosin ring (CAR) at the budding site. Epistasis analyses indicated that Rho5-GTP is also involved in glucose signaling mediated by the small GTPases Ras1/2 and Gpa2, whose signal is further transmitted to activate the protein kinase A (PKA). Other nutrient signals are mediated by TORC1 (target of rapamycin complex 1) through the AKT-related kinase Sch9. Both PKA and Sch9 inhibit the protein kinase Rim15, which controls transcription factors involved in glucose and general stress signaling. PM = plasma membrane, NM = nuclear membrane.

Human Rac1 and yeast Rho5 have been implicated in many different signaling pathways, being activated by a variety of hormones, nutrients, and extracellular stress conditions. Figure 3 presents a general overview of the intracellular distribution and basic physiological impacts of the GTPases in both organisms, with similar protein functions depicted in the same color code. For human Rac1, the figure summarizes data obtained from many different tissues, physiological conditions, and disease phenotypes. Some of these will be discussed briefly in the following, highlighting features that are conserved in yeast Rho5. Moreover, it should be noted that Rac1 has two homologs in humans, which may overlap in function, are expressed in tissue-specific manners, and have been implicated in different forms of cancers, neurological diseases, and as pharmaceutical drug targets (see [2,6,16,17] and references therein). As stated above, this review concentrates on Rac1.

### 3.1. Cell Motility and Plasticity

The role of Rac1 attached to the plasma membrane is best described for its effect on cell motility through its influence on actin dynamics and the formation of membrane protrusions like lamellipodia and filopodia (Figure 3a; [49]). There, the GTPase mediates cell migration by activating the WASP family verprolin-homologous (WAVE) complex (reviewed in [50]). This is, for instance, essential in embryonic development, as demonstrated by the lethality of endothelial-specific knockouts in mice, attributed to the lack of proper cell movement during gastrulation [51]. On the contrary, increased cell motility due to hyperactivation of Rac1—by drugs, point mutations, or the expression of the Rac1b splice variant—can promote the spreading of different cancers through metastasis [52,53]. This is further facilitated by Rac1-dependent activation of metalloproteases, which help invade the extracellular matrix of target tissues [9,20,23,54].

Rac1-mediated remodeling of the actin cytoskeleton is also required for the regulation of neuronal plasticity through activation by its GEF Kalirin-7 [55]. In fact, the modulation of spine morphology depending on Rac1 activity has been related to fear and pain memory [56], depression [57], and even addiction to cocaine [58]. For more detailed information on neurological disorders related to Rac1, two excellent reviews may be consulted [59,60]. As yeast cells neither are motile nor show morphological plasticity independent of cell wall remodeling, the relation of Rho5 to actin organization is basically restricted to the actomyosin ring during cytokinesis, as discussed in Section 3.2.

### 3.2. Rac1 in Cytokinesis

In addition to changes mediated by actin remodeling, non-muscle myosin II was shown to bind to Rho GEFs of the Dbl family and activate Rac1, RhoA, and Cdc42, providing a connection to cytokinesis (Figure 3a; [61]). The three GTPases are then further regulated at the cleavage furrow to mediate actomyosin ring constriction, with RhoA being activated at this site, and Rac1 specifically inhibited by one of its GAPs [62,63].

Reminiscent of these observations, a participation of Rho5 and its dimeric GEF in yeast cytokinesis was suggested by morphological defects in *Klrho5* deletion mutants of the milk yeast *Kluyveromyces lactis*, leading to elongated bud scars after cell division [29]. Indeed, Rho5 colocalizes with the contractile actomyosin ring (CAR) at early stages of cytokinesis, and the morphological defects in the deletion mutants can be efficiently suppressed by a hyper-active Cdc42 variant, in analogy with the coordinated regulation of the small GTPases in mammalian cells (Figure 3b; [29,64]). While a participation of Rho5 in septum formation is also supported by data from *A. gossypii* [65], it should be noted that similar budding defects cannot be observed in *S. cerevisiae rho5* deletion mutants and that actin polymerization is only moderately affected in mutants lacking the Dck1 subunit of the dimeric GEF, as indicated by a reduced number of actin patches [66]. Thus, a functional homology between Rac1 and Rho5 with regard to actin dynamics may be primarily restricted to CAR constriction.

### 3.3. Plasma Membrane Receptors and Signaling Cascades

Signaling to Rac1 can also be triggered by membrane-spanning cadherins or integrin receptors, which detect cell–cell contact or perturbations in the extracellular matrix (ECM), respectively [67,68,69,70]. Integrins recruit a multimeric complex which includes focal adhesion kinases (FAKs), culminating in the activation of the phospholipid kinase PI3K (Figure 3a; [71,72,73]). Rac1 GEFs can also be activated by cadherins. Besides promoting actin polymerization, both signaling pathways lead to the activation of a MAPK cascade, with ERK as the downstream kinase, which further activates PI3K (reviewed in [9,50]). An evolutionary conservation of this Rac1 function is suggested by the fact that Rho5 was first described as a negative regulator of CWI signaling in *S. cerevisiae*, which culminates in the downstream MAPK kinase Slt2 (Figure 3b; [43]). Moreover, a small family of mechanosensors conserved within yeast plasma membranes can detect cell surface perturbations and activate the CWI pathway, thus representing functional equivalents of mammalian integrins [74,75,76,77]. If and how these sensors are involved in the modulation of Rho5 activity remains to be determined, as they rather interact with Rom2, the GEF for another small GTPase, Rho1, to trigger cell wall integrity (CWI) signaling [78,79].

In addition to signaling by integrins and cadherins, other major ways to activate Rac1 are initiated by receptor tyrosine kinases (RTKs). Upon ligand binding (such as of growth hormones), they mediate the activation of the small Ras GTPase, which in turn triggers the ERK MAPK cascade (Figure 3a; [80]). Ras-GTP further promotes PI3K activity and thereby the activation of Rac1 by its GEF P-Rex1 [81]. The latter is also targeted by protein kinase A (PKA), linking G-protein-coupled receptor (GPCR) signaling through cAMP to Rac1 [82]. More directly, Rac1-GTP can interact with PKA to enhance the activation of the downstream ERK MAPK cascade ([83]. A prominent example of GPCR-mediated activation of Rac1 through PI3K is signaling by the chemokine receptor CXCR4 [84,85].

With regard to glucose signaling, the insulin receptor is engaged by its membrane-spanning adaptor proteins, with PI3K as a downstream target. Rac1 activation then results in the increased delivery of vesicles carrying the glucose transporter GLUT4 to the plasma membrane [86,87]. This promotion of glucose transport is mediated in collaboration with another PI3K signaling branch, leading to the activation of protein kinase B (AKT; Figure 3a). AKT signaling also controls glycogen and protein synthesis, as well as playing a role in preventing apoptosis.

The participation of yeast Rho5 in glucose signaling is suggested by its translocation to the mitochondria upon starvation ([66]; Figure 3b). Genetic analyses support this finding, as *rho5* deletions display strong growth defects in conjunction with cells lacking components in glucose signaling, such as Gpr1 and Gpa2, and are synthetically lethal with *sch9* deletions, lacking a protein kinase targeted by the yeast TOR complex [66]. The Ras–cAMP–PKA pathway, especially, the Rim15 protein kinase, seems to transmit signals to transcriptional regulators, influencing nuclear gene expression.

## 4. Oxidative Stress Response, Mitophagy, and Apoptosis

**Figure 4:**
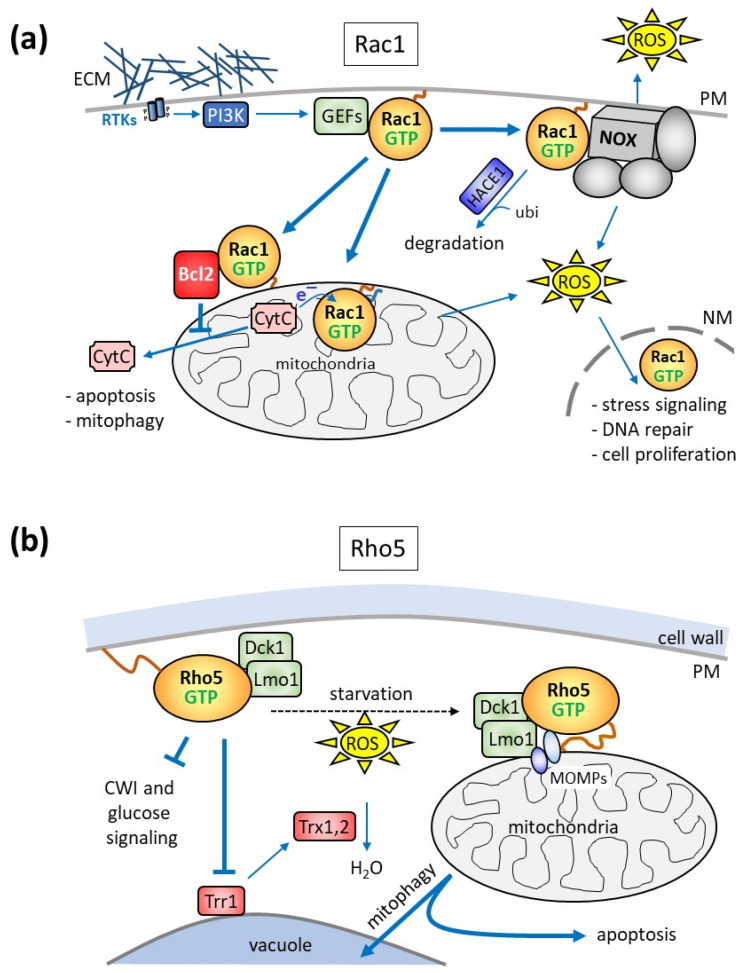
Schematic representation of the intracellular distribution and roles of human Rac1 (**a**) and yeast Rho5 (**b**) in mitophagy and apoptosis. (**a**) Rac1 activation by receptor tyrosine kinases (RTKs) and PI3K, as described above (see Figure 3 and its legend for abbreviations), promotes ROS production by acting as a subunit of NADP-dependent oxidase (NOX) complexes. In macrophages, this serves as a defense mechanism against pathogens by increasing extracellular ROS concentrations, whereas in other tissues, cytosolic ROS triggers the nuclear responses indicated. Rac1-GTP action is diminished by its proteosomal degradation, initiated by the ubiquitin ligase HACE1. Activated Rac1 also interacts with the anti-apoptotic Bcl2 protein to prevent apoptosis. Moreover, palmitoylated Rac1 is imported into mitochondria and serves as an electron receptor for cytochrome C (CytC). Holo-cytochrome C triggers apoptosis when released into the cytosol. In other tissues, Rac1 interaction with mitochondria may also promote mitophagy and apoptosis. The mitochondrial production of reactive oxygen species (ROS) can itself lead to mitochondrial damage and mitophagy and triggers signaling cascades leading to the nuclear import of Rac1 and the modulation of gene expression. (**b**) Yeast Rho5 is a negative regulator of cell wall integrity (CWI) and glucose signaling, as shown in Figure 3. In addition, starvation or exposure to oxidative stress (ROS) triggers the rapid translocation of the GTPase and its dimeric GEF to the mitochondrial surface, indicated by the dotted black arrow, where it triggers mitophagy and apoptosis. The recruitment of Rho5/Dck1/Lmo1 is probably mediated by certain mitochondrial outer membrane proteins (MOMPs; see text for further details). Another way of influencing the response to oxidative stress is mediated by the inhibition of thioredoxin reductase (Trr1), which provides the reduction equivalents for the detoxification of ROS by thioredoxins (Trx1 and Trx2).

Apart from the physiological functions presumably exerted following their activation at the plasma membrane described above, accumulating evidence suggests that Rac1 and Rho5 participate in the production of reactive oxygen species (ROS) and the regulation of mitophagy and apoptosis. This can be achieved either by promoting an oxidase activity or when the GTPases are translocated to mitochondria in both mammals and yeast (Figure 4; [12,13,41]). These relations have been more extensively studied for mammalian Rac1 in the contexts of cancer development, immune response, brain functions, and diabetes, with some striking similarities observed for its fungal homologues, as outlined below.

### 4.1. ROS Generation and Function

Rac1 has been proposed to be a regulatory subunit of some NADPH oxidase (NOX) complexes, which generate reactive oxygen species (ROS; Figure 4a; [88,89,90,91,92]). NOX complexes are composed of the central, name-giving catalytic subunit with several membrane-spanning domains and up to six proteins (mostly designated by their molecular weight and the term “phox” used for their first description in phagocyte oxidases, e.g., p22^phox^), either carrying membrane-spanning domains or being associated from the cytosol [93]. The NOX family comprises seven members, with NOX1, NOX2, and NOX3, residing in caveolar, vesicle, and plasma membranes, respectively. In contrast to these three, NOX4, located in the mitochondrial outer membrane, and NOX5 to NOX7, do not include a Rac regulatory subunit [93].

Although first discovered as a mechanism to combat bacterial pathogens in macrophages, NOX-dependent ROS generation triggered by Rac1 or its homologue Rac2 affects cell proliferation, apoptosis, and senescence [91,94,95,96,97,98,99] (Figure 4a). This route of ROS generation can also be engaged by growth factor and integrin signaling [100,101] and plays an important role in neuron development and neurodegenerative diseases [102,103,104]. Moreover, it was found to be a leading cause and putative therapeutic target in diabetes-related blindness [105]. Rac1/NOX signaling is tightly controlled by the ubiquitin/proteasome-mediated degradation of the GTPase, as a decreased activity of the corresponding HACE1 ubiquitin ligase in eukaryotic model organisms or in tumor cell lines promotes cancer development [106].

Interestingly, Rac1 homologues in different filamentous fungi were found to regulate hyphal growth, actin dynamics, and defense against pathogens through NOX/ROS, indicating that this function is conserved in fungi (reviewed in [41,107]). In baker’s yeast, Yno1/Aim14 has been identified as a putative NOX homologue and shown to reside primarily in the perinuclear endoplasmic reticulum [108]. However, possible interactions with Rho5 in oxidative stress response and apoptosis have not yet been investigated.

Rather, Rho5 inhibits the thioredoxin reductase Trr1 and thus reduces the conversion of ROS into water by the yeast thioredoxins (Figure 4b; [109]). Consequently, *rho5* deletion mutants show increased capacity for detoxification of ROS and a decreased rate of apoptosis.

### 4.2. Association of Rac1/Rho5 with Mitochondria

Rac1 was already found in 1995 in mitochondrial fractions derived from the kidney cortex of rats, which were enriched neither in RhoA nor in Cdc42 [110]. However, this finding only gained considerable attention more than 15 years later, when a physical interaction between Rac1 and Bcl-2 was reported at the mitochondrial surface in a human cancer cell line, which stabilized the anti-apoptotic activity of the latter protein (Figure 4a; [111]). This notion was further supported by the discovery of the interaction of Rac1 and Bcl-2 in mitochondrial preparations from bovine brain, where the two proteins were found in a complex with the sigma-1 receptor (SigR1; associated with memory functions and drug dependence) and the inositol 1,4,5-trisphosphate receptor [112]. The authors concluded that the trimeric signaling complex contributes to neuroplasticity and generates a mild oxidative stress, protecting cells from autophagy and apoptosis. In other tissues, the complex may enhance rather than prevent apoptosis, as inhibition or depletion of Rac1 in mouse models of diabetes promoted survival and alleviated mitochondria-related oxidative stress [113]. In any case, mitochondrial dysfunctions leading to increased ROS production at variable degrees are believed to cause mitophagy and apoptosis. This then leads to DNA damage, induction of repair systems, and changes in nuclear gene expression mediated by various signaling pathways (reviewed in [12]).

In addition to the Rac1 functions executed by interactions at the mitochondrial surface, the GTPase can translocate into mitochondria and contribute to hydrogen peroxide production, as shown for alveolar macrophages from patients with pulmonary fibrosis [114]. Mitochondrial import requires geranylgeranylation at the CAAX-box cysteine residue (Cys189), whereas Cys178 serves as an electron acceptor from cytochrome C. Parallel studies in zebrafish and mice endothelial cells suggested that mitochondrial import is mediated by the translocase of the outer membrane (i.e., the TOM complex), and depletion of a small subunit enhances the import of Rac1, with the concomitant increase in ROS production, causing defects in angiogenesis and cerebral malformations [115].

Strong evidence for a role of yeast Rho5 in mitochondrial turnover was provided by the observation that the GTPase and its activating dimeric GEF Dck1/Lmo1 rapidly translocated to mitochondria upon application of oxidative stress (Figure 4b; [38]). The respective deletion mutants displayed hyper-resistance towards hydrogen peroxide and a reduced apoptotic cell death. Together with the observed decrease in mitophagy, this strongly suggested that wild-type Rho5 is required to trigger both mitophagy and apoptosis [38,109,116,117]. This notion was further supported when Atg21, a component of the mitophagic pathway, was found to interact with GTP-bound Rho5 in a genome-wide screen [118]. Another component appearing in this screen was the mitochondrial outer membrane (MOMP) protein Msp1. It is to be expected that other MOMPs, such as Alo1 and Fun14, may be required for the efficient translocation of Rho5 to mitochondria under oxidative stress, together with Msp1. A putative yeast homologue of the mammalian sigma-1 receptor described above is Erg2, whose interaction with Rho5 has not been investigated [119]. Yet, given that Erg2 resides in the endoplasmic reticulum and that it is not listed in any of the large-scale screens in relation to Rho5 according to the *Saccharomyces* genome database (https://www.yeastgenome.org, accessed on 15 December 2023), an important functional interaction seems unlikely.

With regard to other factors influencing the intracellular distribution of Rho5, its translocation to mitochondria under oxidative stress requires the presence of both of its GEF subunits, whereas Dck1 and Lmo1 can be recruited independently of each other [38,120]. Apart from these findings and the need for its C-terminal modification, the exact mode of Rho5 recruitment to mitochondria remains elusive [30,120]. Evidence from studies on trapping Rho5 or its GEF subunits to different membranes indicates that in order to exert its function in oxidative stress response, the GTPase needs to be activated at the plasma membrane prior to its translocation to mitochondria. However, we assume that in analogy with the wealth of data connecting Rac1 localization in mammalian cells to its association with different GEFs, Dck1/Lmo1 may be a major guiding device. Further investigations on the spatiotemporal distribution and post-translational modifications of Rho5 under different physiological conditions to address this issue are in progress.

Finally, the mitochondrial import or an intramitochondrial function of yeast Rho5 seems unlikely, since the electron-accepting Cys178 residue of Rac1 is not conserved in Rho5. Moreover, the rapid association with mitochondria under oxidative stress and glucose starvation is completely reversible, once the stress is alleviated. This would also argue against the translocation of the GTPase across the mitochondrial membranes, which would be expected to be more permanent.

## 5. Other Regulatory Networks

The role in cell cycle regulation exerted by Rac1 in mammalian cells does not seem to be conserved for yeast Rho5. Specifically, Rac1 can translocate to the nucleus upon its phosphorylation by ERK (extracellular signal receptor kinase) and participate in transcriptional regulation (Figure 3a; [121]). More global effects on Rac1-dependent gene expression are achieved through its activation of different mitogen-activated protein kinase (MAPK) cascades, with the phosphorylation of transcription factors by their downstream kinases ERK, p38, and JUNK (reviewed in [40]). Frequently, this is enhanced by the activation of the upstream p21-activated kinase (PAK) by Rac1 [122,123,124] (see [12] for an extensive review).

Other functions of Rac1 in the regulation of gene expression upon its translocation to the nucleus and to other endomembranes will not be discussed here in more detail, as they also do not seem to be conserved in yeast Rho5 and have been extensively reviewed elsewhere [44,125]. Likewise, an interactive network with other small GTPases as evident from numerous studies on mammalian Rac1 (recently summarized in [126,127,128,129]) has barely been experimentally tackled for yeast Rho5, so far.

## 6. Conclusions and Future Perspectives

Despite the huge evolutionary distance between yeast and humans, we here summarized some striking similarities in the signaling networks and physiological functions of the small GTPases Rho5 and Rac1. Thus, both are connected to MAPK signaling pathways (CWI in yeast, and PAK/ERK in humans). On the other hand, the role in the regulation of actin dynamics originally assigned to Rac1 seems to be only marginally conserved in yeast, mainly related to actomyosin ring constriction during cytokinesis. A concerted action with other small GTPases, as observed for Rac1, has not yet been investigated for yeast Rho5, including the possibility of the existence of positive feedback loops with its activating dimeric GEF. More recently, the involvement of Rac1 in the generation of ROS (both in the cytosol and by mitochondria) has drawn considerable attention and shown to mediate mitophagy and apoptosis, e.g., in neurons and endothelial cells. The latter processes are also controlled by Rho5 activity in yeast, indicating at least a functional conservation.

Yeast systems provide an advantage over mammalian models like mice and rats, in that they can easily be tackled by genetic manipulations. For example, one approach we are currently following is to look for more in vivo interactions of Rho5 and its dimeric GEF employing biotinylation by the TurboID system [130]. The new interactors thus identified will provide hints on both the upstream activators and the downstream effectors mediating the different physiological functions.

Yeasts may also serve to study specific interactions of Rac1 observed in mammalian cells, as demonstrated by studies on annexin–tau interactions related to Alzheimer’s disease [131,132]. Given the synthetic lethality of *rho5 sch9* deletions and its rescue by a chimeric Rac1 construct carrying the LYSE of Rho5, high-throughput screens could be performed, e.g., for nucleotide analogues specifically targeting Rac1 [9,133,134]. More complex approaches could also involve the reconstitution of functional complexes in yeast, like Rac1/NOX, to study their interactions and their use as possible drug targets, as done, for instance for the PI3K–PTEN–Akt signaling pathway [135].

Despite the considerable knowledge on Rac1 function in human health and disease that has been gathered over the past three decades, there is still much more to be discovered, with the “awesome power of yeast genetics” [136,137] at research service.

## Supplementary Materials

The following supporting information can be downloaded at: https://www.mdpi.com/article/10.3390/cells13060472/s1, Figure S1: Alignment of different Rho5 homologues from fungi and humans.
