# Supplementary material for: Functional Conservation of the Small GTPase Rho5/Rac1—A Tale of Yeast and Men
Source: Cells. 2024 Mar 7;13(6):472. doi: 10.3390/cells13060472 (PMC10969153; doi:10.3390/cells13060472)
Supplement: Supplementary file 1 [file cells-13-00472-s001.zip › cells-2882787-supplementary.pdf]

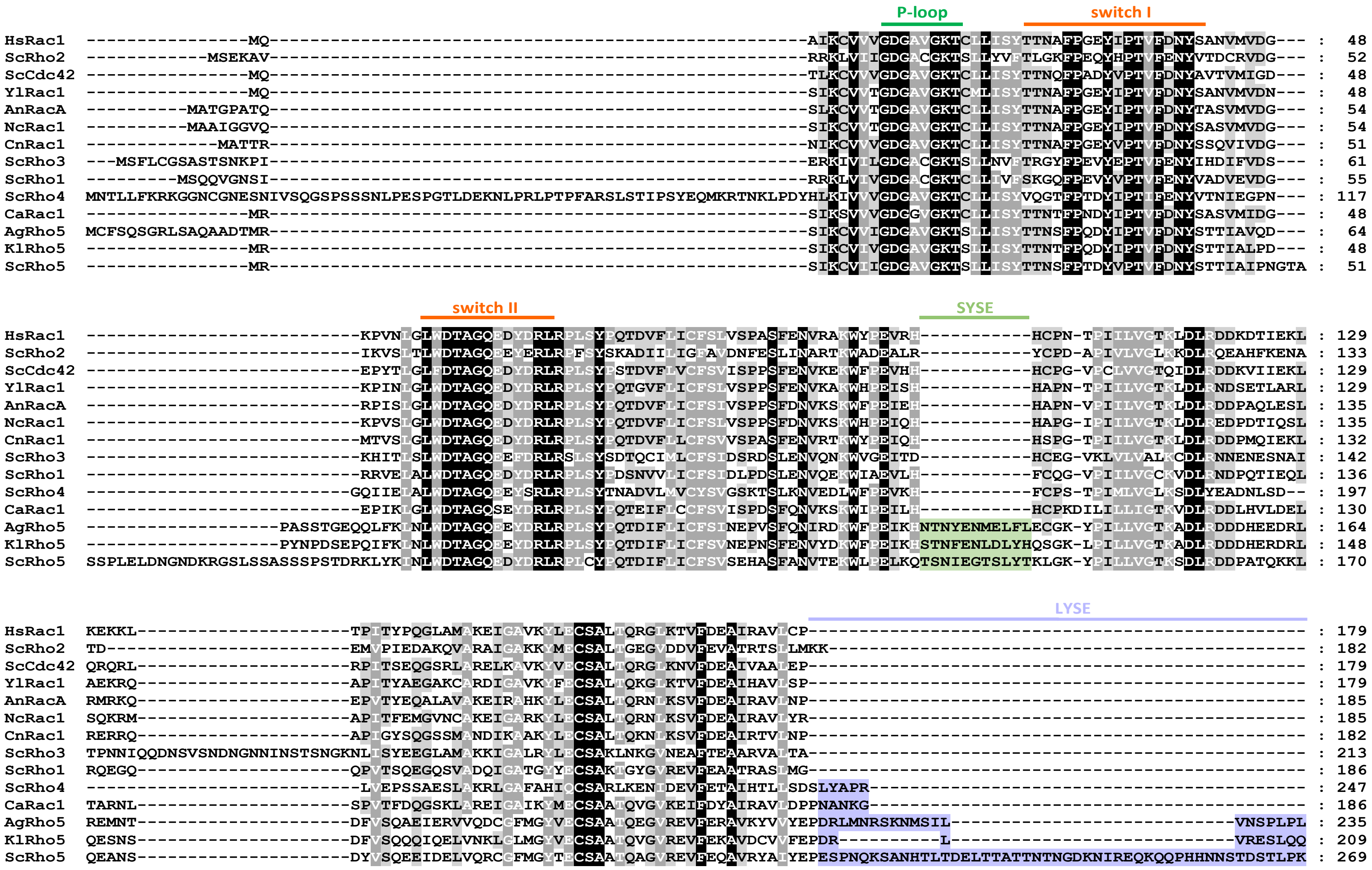


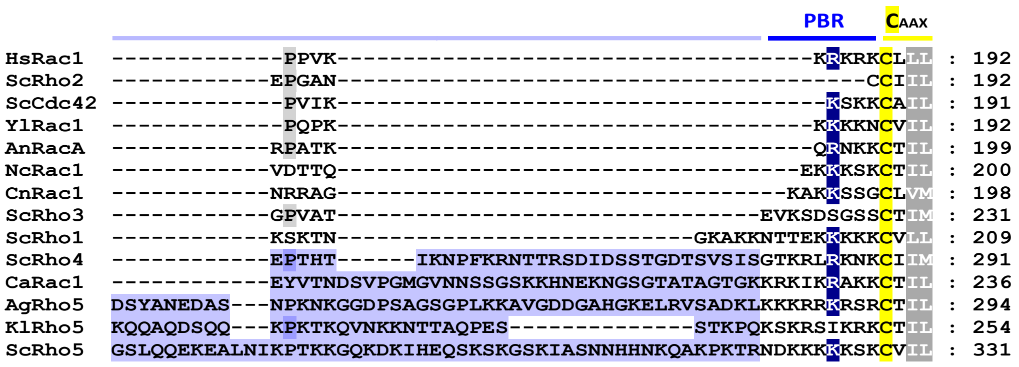


**Supplementary Figure S1:** Alignment of the sequences of Rac1 with homologoues from different yeasts and other fungi. Programms and parameters used are described in the legend of Figure 1a in the main text. GTPases from different organisms and their protein database accession numbers are (available at https://www.uniprot.org): Hs = *Homo sapiens* (P63000); Sc = *Saccharomyces cerevisiae*, Rho2 (P06781); Cdc42 (P19073); Rho3 (Q00245); Rho4 (Q00246); Rho5 (P53879); Kl = *Kluyveromyces lactis* (A0A5P2U790); Yl = *Yarrowia lipolytica* (Q9P8X2); An = *Aspergillus nidulans* (Q5B3Y7); Nc = *Neurospora crassa* (Q7RW05); Cn = *Cryptococcus neoformans* (A0A024QYS1); Ca = *Candida albicans* (A0A1D8PDV5); Ag = *Ashbya gossypii* (Q75E12).
